# 4-[1-Acetyl-3-(4-methoxy­phen­yl)-2-pyrazolin-5-yl]phenol

**DOI:** 10.1107/S1600536809043785

**Published:** 2009-10-28

**Authors:** Xue Bai, Hua-feng Chen, Kuan Zhang, Ying Li, Shu-fan Yin

**Affiliations:** a College of Chemistry, Sichuan University, Chengdu 610064, People’s Republic of China

## Abstract

In the title compound, C_18_H_18_N_2_O_3_, the dihedral angle formed by the benzene rings is 71.75 (4)°. In the crystal structure, centrosymmetrically related mol­ecules are linked into dimers by inter­molecular O—H⋯O hydrogen bonds and π–π stacking inter­actions with centroid–centroid distances of 3.5511 (6) Å.

## Related literature

For the biological activity of 2-pyrazoline derivatives, see: Grimm *et al.* (2009 ▶). For the synthesis and crystal structure of 2-pyrazoline derivatives, see: Chen *et al.* (2009 ▶); Li *et al.* (2008 ▶); Humaira *et al.* (2008 ▶); Shoman *et al.* (2009 ▶). 
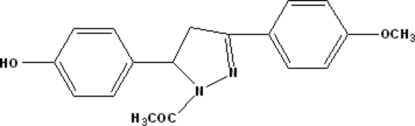


## Experimental

### 

#### Crystal data

C_18_H_18_N_2_O_3_
*M* *_r_* = 310.34
Monoclinic, 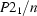

*a* = 8.7037 (17) Å
*b* = 15.673 (3) Å
*c* = 11.096 (2) Å
β = 100.31 (3)°
*V* = 1489.2 (5) Å^3^
*Z* = 4
Mo *K*α radiation
μ = 0.10 mm^−1^
*T* = 113 K
0.28 × 0.25 × 0.23 mm

#### Data collection

Rigaku Saturn CCD area-detector diffractometer
Absorption correction: multi-scan (*CrystalClear*; Rigaku/MSC, 2005 ▶) *T*_min_ = 0.974, *T*_max_ = 0.978
12107 measured reflections
3542 independent reflections
2857 reflections with *I* > 2σ(*I*)
*R*_int_ = 0.035

#### Refinement

*R*[*F* ^2^ > 2σ(*F* ^2^)] = 0.038
*wR*(*F* ^2^) = 0.107
*S* = 1.07
3542 reflections
211 parameters
H-atom parameters constrained
Δρ_max_ = 0.28 e Å^−3^
Δρ_min_ = −0.22 e Å^−3^

### 

Data collection: *CrystalClear* (Rigaku/MSC, 2005 ▶); cell refinement: *CrystalClear*; data reduction: *CrystalClear*; program(s) used to solve structure: *SHELXS97* (Sheldrick, 2008 ▶); program(s) used to refine structure: *SHELXL97* (Sheldrick, 2008 ▶); molecular graphics: *ORTEP-3 for Windows* (Farrugia, 1997 ▶); software used to prepare material for publication: *SHELXL97*.

## Supplementary Material

Crystal structure: contains datablocks global, I. DOI: 10.1107/S1600536809043785/rz2374sup1.cif

Structure factors: contains datablocks I. DOI: 10.1107/S1600536809043785/rz2374Isup2.hkl

Additional supplementary materials:  crystallographic information; 3D view; checkCIF report

## Figures and Tables

**Table 1:** Hydrogen-bond geometry (Å, °)

*D*—H⋯*A*	*D*—H	H⋯*A*	*D*⋯*A*	*D*—H⋯*A*
O1—H1⋯O2^i^	0.84	1.87	2.7117 (13)	175

Symmetry code: (i) 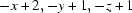.

**Fig. 1.**
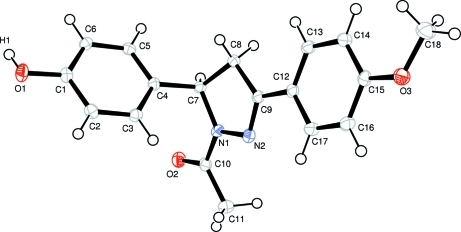
The molecular structure of the title compound, with displacement ellipsoids drawn at the 30% probability level.

## supplementary crystallographic information

### Comment

The 2-pyrazoline ring system has attracted significant interest in organic and medicinal chemistry over the past several decades. Scaffolds containing the 2-pyrazoline (4,5-dihydropyrazole) heterocycle have demonstrated a wide range of biological activity, including anticancer activity through the inhibition of kinesin spindle protein, CB1 receptor antagonism for obesity, monoamine oxidase inhibition for depression, and a host of other antibacterial, antiviral, and anti-inflammatory activities (Grimm *et al.*, 2009). Some crystal structure of pyrazoline derivatives have been recently reported (Chen *et al.*, 2009; Li *et al.*, 2008). The synthesis and characterization of pyrazoline derivatives was also reported (Humaira *et al.*, 2008; Shoman *et al.*, 2009).

In the molecule of the title compound (Fig. 1), the five-membered 2-pyrazoline ring assumes an envelope conformation, with atom C7 displaced by 0.2690 (11) Å from the mean plane of the N1/N2/C8/C9 atoms. The benzene rings form a dihedral angle of 108.25 (4)°. In the crystal structure, centrosymmetrically related molecules are linked into dimers by intermolecular O—H···.O hydrogen bonds (Table 1) and by a π–π stacking interaction involving the C1–C6 aromatic rings, with a centroid-to-centroid distance of 3.5511 (6) Å.

### Experimental

A mixture of 4'-methoxy-4-hydroxychalcone (0.64 g,2.5 mmol) and hydrazine hydrate (1 ml) in acetic acid (15 ml) was refluxed for 2 h. The reaction mixture was then cooled at room temperature, and poured into ice-cold water. The light yellow solid obtained was filtered, washed with water, dichloromethane, and dried. Colourless crystals suitable for X-ray analysis were obtained by slow evaporation of an acetone/dichloromethane (3:1 *v*/*v*) solution at room temperature.

### Refinement

H atoms were positioned geometrically (C—H = 0.93–0.98 Å, O—H = 0.82 Å) and refined using a riding model, with *U*_iso_(H) = 1.2*U*_eq_(C) or 1.5*U*_eq_(C, O) for methyl and hydroxy H atoms.

### Crystal data

**Table:**

C_18_H_18_N_2_O_3_	*F*(000) = 656
*M**_r_* = 310.34	*D*_x_ = 1.384 Mg m^−^^3^
Monoclinic, *P*2_1_/*n*	Mo *K*α radiation, λ = 0.71073 Å
Hall symbol: -P 2yn	Cell parameters from 4953 reflections
*a* = 8.7037 (17) Å	θ = 1.9–27.9°
*b* = 15.673 (3) Å	µ = 0.10 mm^−^^1^
*c* = 11.096 (2) Å	*T* = 113 K
β = 100.31 (3)°	Block, colourless
*V* = 1489.2 (5) Å^3^	0.28 × 0.25 × 0.23 mm
*Z* = 4	

### Data collection

**Table:**

Rigaku Saturn CCD area-detector diffractometer	3542 independent reflections
Radiation source: rotating anode	2857 reflections with *I* > 2σ(*I*)
confocal	*R*_int_ = 0.035
Detector resolution: 7.31 pixels mm^-1^	θ_max_ = 27.9°, θ_min_ = 2.3°
ω and φ scans	*h* = −11→10
Absorption correction: multi-scan (*CrystalClear*; Rigaku/MSC, 2005)	*k* = −20→20
*T*_min_ = 0.974, *T*_max_ = 0.978	*l* = −10→14
12107 measured reflections	

### Refinement

**Table:**

Refinement on *F*^2^	Primary atom site location: structure-invariant direct methods
Least-squares matrix: full	Secondary atom site location: difference Fourier map
*R*[*F*^2^ > 2σ(*F*^2^)] = 0.038	Hydrogen site location: inferred from neighbouring sites
*wR*(*F*^2^) = 0.107	H-atom parameters constrained
*S* = 1.07	*w* = 1/[σ^2^(*F*_o_^2^) + (0.0618*P*)^2^ + 0.155*P*] where *P* = (*F*_o_^2^ + 2*F*_c_^2^)/3
3542 reflections	(Δ/σ)_max_ < 0.001
211 parameters	Δρ_max_ = 0.28 e Å^−^^3^
0 restraints	Δρ_min_ = −0.22 e Å^−^^3^

### Special details

**Table:**

Geometry. All e.s.d.'s (except the e.s.d. in the dihedral angle between two l.s. planes) are estimated using the full covariance matrix. The cell e.s.d.'s are taken into account individually in the estimation of e.s.d.'s in distances, angles and torsion angles; correlations between e.s.d.'s in cell parameters are only used when they are defined by crystal symmetry. An approximate (isotropic) treatment of cell e.s.d.'s is used for estimating e.s.d.'s involving l.s. planes.
Refinement. Refinement of *F*^2^ against ALL reflections. The weighted *R*-factor *wR* and goodness of fit *S* are based on *F*^2^, conventional *R*-factors *R* are based on *F*, with *F* set to zero for negative *F*^2^. The threshold expression of *F*^2^ > 2σ(*F*^2^) is used only for calculating *R*-factors(gt) *etc*. and is not relevant to the choice of reflections for refinement. *R*-factors based on *F*^2^ are statistically about twice as large as those based on *F*, and *R*- factors based on ALL data will be even larger.

### Fractional atomic coordinates and isotropic or equivalent isotropic displacement parameters (Å^2^)

**Table:**

	*x*	*y*	*z*	*U*_iso_*/*U*_eq_	
O1	0.94883 (10)	0.38855 (6)	0.70894 (7)	0.0229 (2)	
H1	1.0433	0.3749	0.7160	0.034*	
O2	0.75176 (10)	0.66339 (5)	0.27990 (7)	0.0225 (2)	
O3	−0.11026 (10)	0.27148 (5)	−0.04411 (8)	0.0231 (2)	
N1	0.58975 (11)	0.55520 (6)	0.21083 (8)	0.0167 (2)	
N2	0.43790 (11)	0.52531 (6)	0.17203 (8)	0.0171 (2)	
C1	0.89186 (13)	0.40973 (7)	0.59032 (10)	0.0168 (2)	
C2	0.75202 (13)	0.45422 (7)	0.56538 (10)	0.0182 (2)	
H2	0.6974	0.4675	0.6298	0.022*	
C3	0.69165 (13)	0.47942 (7)	0.44593 (10)	0.0168 (2)	
H3	0.5958	0.5099	0.4295	0.020*	
C4	0.77000 (13)	0.46058 (7)	0.35034 (9)	0.0151 (2)	
C5	0.90875 (13)	0.41512 (7)	0.37684 (10)	0.0163 (2)	
H5	0.9631	0.4015	0.3123	0.020*	
C6	0.97009 (13)	0.38908 (7)	0.49508 (10)	0.0164 (2)	
H6	1.0646	0.3574	0.5111	0.020*	
C7	0.70776 (13)	0.48686 (7)	0.21887 (10)	0.0159 (2)	
H7	0.7957	0.5053	0.1780	0.019*	
C8	0.61163 (13)	0.41600 (7)	0.14272 (10)	0.0171 (2)	
H8A	0.6386	0.4113	0.0600	0.021*	
H8B	0.6277	0.3600	0.1843	0.021*	
C9	0.44632 (13)	0.44749 (7)	0.13665 (9)	0.0160 (2)	
C10	0.61741 (14)	0.63762 (7)	0.24368 (9)	0.0179 (2)	
C11	0.47757 (15)	0.69509 (8)	0.23326 (11)	0.0233 (3)	
H11A	0.5121	0.7537	0.2534	0.035*	
H11B	0.4107	0.6759	0.2903	0.035*	
H11C	0.4186	0.6931	0.1493	0.035*	
C12	0.30403 (13)	0.39938 (7)	0.09013 (9)	0.0163 (2)	
C13	0.31003 (14)	0.31987 (7)	0.03532 (10)	0.0183 (2)	
H13	0.4088	0.2956	0.0306	0.022*	
C14	0.17471 (14)	0.27499 (7)	−0.01279 (10)	0.0191 (2)	
H14	0.1812	0.2213	−0.0512	0.023*	
C15	0.03036 (13)	0.30954 (7)	−0.00400 (10)	0.0180 (2)	
C16	0.02255 (14)	0.38880 (7)	0.05220 (10)	0.0205 (3)	
H16	−0.0763	0.4123	0.0584	0.025*	
C17	0.15651 (14)	0.43316 (8)	0.09871 (10)	0.0199 (2)	
H17	0.1494	0.4869	0.1369	0.024*	
C18	−0.11105 (16)	0.19636 (9)	−0.11600 (13)	0.0320 (3)	
H18A	−0.0643	0.2087	−0.1881	0.048*	
H18B	−0.2188	0.1769	−0.1424	0.048*	
H18C	−0.0506	0.1516	−0.0671	0.048*	

### Atomic displacement parameters (Å^2^)

**Table:**

	*U*^11^	*U*^22^	*U*^33^	*U*^12^	*U*^13^	*U*^23^
O1	0.0209 (5)	0.0314 (5)	0.0155 (4)	0.0013 (4)	0.0014 (3)	0.0053 (3)
O2	0.0226 (5)	0.0219 (4)	0.0217 (4)	−0.0016 (3)	0.0006 (3)	0.0008 (3)
O3	0.0177 (4)	0.0242 (4)	0.0269 (4)	−0.0011 (3)	0.0027 (3)	−0.0048 (3)
N1	0.0146 (5)	0.0173 (5)	0.0179 (4)	0.0021 (4)	0.0021 (4)	0.0011 (3)
N2	0.0161 (5)	0.0191 (5)	0.0158 (4)	0.0003 (4)	0.0021 (4)	0.0015 (4)
C1	0.0174 (6)	0.0167 (5)	0.0158 (5)	−0.0046 (4)	0.0017 (4)	0.0015 (4)
C2	0.0174 (6)	0.0216 (6)	0.0165 (5)	−0.0023 (4)	0.0058 (4)	−0.0007 (4)
C3	0.0133 (5)	0.0182 (5)	0.0190 (5)	0.0000 (4)	0.0035 (4)	−0.0002 (4)
C4	0.0156 (5)	0.0144 (5)	0.0150 (5)	−0.0021 (4)	0.0022 (4)	0.0001 (4)
C5	0.0174 (6)	0.0155 (5)	0.0168 (5)	−0.0007 (4)	0.0054 (4)	−0.0013 (4)
C6	0.0141 (6)	0.0147 (5)	0.0201 (5)	−0.0002 (4)	0.0020 (4)	0.0008 (4)
C7	0.0154 (5)	0.0172 (5)	0.0155 (5)	0.0033 (4)	0.0040 (4)	0.0002 (4)
C8	0.0170 (6)	0.0193 (5)	0.0148 (5)	0.0024 (4)	0.0023 (4)	−0.0011 (4)
C9	0.0177 (6)	0.0187 (5)	0.0118 (5)	0.0037 (4)	0.0030 (4)	0.0018 (4)
C10	0.0229 (6)	0.0177 (5)	0.0134 (5)	0.0007 (5)	0.0037 (4)	0.0021 (4)
C11	0.0261 (6)	0.0176 (5)	0.0260 (6)	0.0033 (5)	0.0046 (5)	−0.0011 (5)
C12	0.0176 (6)	0.0186 (5)	0.0128 (5)	0.0017 (4)	0.0027 (4)	0.0022 (4)
C13	0.0174 (6)	0.0196 (5)	0.0184 (5)	0.0042 (4)	0.0043 (4)	0.0007 (4)
C14	0.0216 (6)	0.0171 (5)	0.0183 (5)	0.0021 (4)	0.0030 (4)	−0.0009 (4)
C15	0.0179 (6)	0.0212 (6)	0.0146 (5)	0.0003 (4)	0.0019 (4)	0.0029 (4)
C16	0.0177 (6)	0.0230 (6)	0.0212 (5)	0.0041 (5)	0.0047 (4)	−0.0009 (4)
C17	0.0210 (6)	0.0202 (5)	0.0188 (5)	0.0041 (5)	0.0040 (4)	−0.0017 (4)
C18	0.0253 (7)	0.0251 (6)	0.0435 (8)	−0.0009 (5)	0.0008 (6)	−0.0114 (6)

### Geometric parameters (Å, °)

**Table:**

O1—C1	1.3619 (13)	C8—C9	1.5112 (15)
O1—H1	0.8400	C8—H8A	0.9900
O2—C10	1.2345 (15)	C8—H8B	0.9900
O3—C15	1.3626 (14)	C9—C12	1.4628 (16)
O3—C18	1.4216 (15)	C10—C11	1.5018 (16)
N1—C10	1.3521 (15)	C11—H11A	0.9800
N1—N2	1.3957 (13)	C11—H11B	0.9800
N1—C7	1.4757 (14)	C11—H11C	0.9800
N2—C9	1.2873 (14)	C12—C13	1.3917 (15)
C1—C2	1.3870 (16)	C12—C17	1.4077 (16)
C1—C6	1.3939 (15)	C13—C14	1.3935 (16)
C2—C3	1.3927 (16)	C13—H13	0.9500
C2—H2	0.9500	C14—C15	1.3875 (16)
C3—C4	1.3915 (15)	C14—H14	0.9500
C3—H3	0.9500	C15—C16	1.3970 (16)
C4—C5	1.3876 (16)	C16—C17	1.3762 (17)
C4—C7	1.5193 (15)	C16—H16	0.9500
C5—C6	1.3865 (15)	C17—H17	0.9500
C5—H5	0.9500	C18—H18A	0.9800
C6—H6	0.9500	C18—H18B	0.9800
C7—C8	1.5474 (16)	C18—H18C	0.9800
C7—H7	1.0000		
			
C1—O1—H1	109.5	N2—C9—C12	120.34 (10)
C15—O3—C18	117.37 (9)	N2—C9—C8	113.68 (10)
C10—N1—N2	121.13 (9)	C12—C9—C8	125.91 (10)
C10—N1—C7	126.07 (10)	O2—C10—N1	120.86 (10)
N2—N1—C7	112.67 (9)	O2—C10—C11	122.43 (10)
C9—N2—N1	108.04 (9)	N1—C10—C11	116.71 (10)
O1—C1—C2	118.04 (10)	C10—C11—H11A	109.5
O1—C1—C6	122.24 (10)	C10—C11—H11B	109.5
C2—C1—C6	119.72 (10)	H11A—C11—H11B	109.5
C1—C2—C3	120.04 (10)	C10—C11—H11C	109.5
C1—C2—H2	120.0	H11A—C11—H11C	109.5
C3—C2—H2	120.0	H11B—C11—H11C	109.5
C4—C3—C2	120.82 (10)	C13—C12—C17	118.18 (11)
C4—C3—H3	119.6	C13—C12—C9	121.34 (10)
C2—C3—H3	119.6	C17—C12—C9	120.47 (10)
C5—C4—C3	118.30 (10)	C12—C13—C14	121.61 (10)
C5—C4—C7	119.33 (9)	C12—C13—H13	119.2
C3—C4—C7	122.36 (10)	C14—C13—H13	119.2
C6—C5—C4	121.65 (10)	C15—C14—C13	119.34 (10)
C6—C5—H5	119.2	C15—C14—H14	120.3
C4—C5—H5	119.2	C13—C14—H14	120.3
C5—C6—C1	119.44 (10)	O3—C15—C14	125.30 (11)
C5—C6—H6	120.3	O3—C15—C16	114.99 (10)
C1—C6—H6	120.3	C14—C15—C16	119.69 (11)
N1—C7—C4	112.31 (9)	C17—C16—C15	120.73 (11)
N1—C7—C8	100.69 (9)	C17—C16—H16	119.6
C4—C7—C8	113.25 (9)	C15—C16—H16	119.6
N1—C7—H7	110.1	C16—C17—C12	120.42 (11)
C4—C7—H7	110.1	C16—C17—H17	119.8
C8—C7—H7	110.1	C12—C17—H17	119.8
C9—C8—C7	101.97 (9)	O3—C18—H18A	109.5
C9—C8—H8A	111.4	O3—C18—H18B	109.5
C7—C8—H8A	111.4	H18A—C18—H18B	109.5
C9—C8—H8B	111.4	O3—C18—H18C	109.5
C7—C8—H8B	111.4	H18A—C18—H18C	109.5
H8A—C8—H8B	109.2	H18B—C18—H18C	109.5
			
C10—N1—N2—C9	174.49 (9)	N1—N2—C9—C8	−2.58 (12)
C7—N1—N2—C9	−9.40 (11)	C7—C8—C9—N2	12.40 (12)
O1—C1—C2—C3	178.11 (10)	C7—C8—C9—C12	−170.70 (9)
C6—C1—C2—C3	−1.14 (16)	N2—N1—C10—O2	178.76 (9)
C1—C2—C3—C4	−0.03 (17)	C7—N1—C10—O2	3.20 (16)
C2—C3—C4—C5	0.80 (16)	N2—N1—C10—C11	−1.45 (14)
C2—C3—C4—C7	179.90 (10)	C7—N1—C10—C11	−177.02 (9)
C3—C4—C5—C6	−0.41 (16)	N2—C9—C12—C13	170.72 (10)
C7—C4—C5—C6	−179.55 (10)	C8—C9—C12—C13	−5.99 (16)
C4—C5—C6—C1	−0.73 (16)	N2—C9—C12—C17	−8.61 (15)
O1—C1—C6—C5	−177.70 (10)	C8—C9—C12—C17	174.68 (10)
C2—C1—C6—C5	1.51 (16)	C17—C12—C13—C14	1.49 (16)
C10—N1—C7—C4	71.36 (13)	C9—C12—C13—C14	−177.86 (10)
N2—N1—C7—C4	−104.52 (10)	C12—C13—C14—C15	−1.19 (16)
C10—N1—C7—C8	−167.87 (10)	C18—O3—C15—C14	−9.49 (16)
N2—N1—C7—C8	16.25 (10)	C18—O3—C15—C16	171.96 (10)
C5—C4—C7—N1	−162.58 (9)	C13—C14—C15—O3	−178.16 (10)
C3—C4—C7—N1	18.32 (15)	C13—C14—C15—C16	0.33 (16)
C5—C4—C7—C8	84.19 (12)	O3—C15—C16—C17	178.82 (10)
C3—C4—C7—C8	−94.91 (12)	C14—C15—C16—C17	0.18 (17)
N1—C7—C8—C9	−15.77 (10)	C15—C16—C17—C12	0.15 (17)
C4—C7—C8—C9	104.33 (10)	C13—C12—C17—C16	−0.96 (16)
N1—N2—C9—C12	−179.67 (8)	C9—C12—C17—C16	178.39 (10)

### Hydrogen-bond geometry (Å, °)

**Table:**

*D*—H···*A*	*D*—H	H···*A*	*D*···*A*	*D*—H···*A*
O1—H1···O2^i^	0.84	1.87	2.7117 (13)	175

Symmetry codes: (i) −*x*+2, −*y*+1, −*z*+1.
